# Effects of Comorbidities on the Elderly Patients with COVID-19: Clinical Characteristics of Elderly Patients Infected with COVID-19 from Sichuan, China

**DOI:** 10.1007/s12603-020-1486-1

**Published:** 2020-10-12

**Authors:** S.-P. Dai, X. Zhao, Jin-hui Wu

**Affiliations:** 1 National Center for Geriatrics Clinical Medicine Research, Department of Geriatrics and Gerontology, West China Hospital, Sichuan University, Chengdu, 610041 China; 2 The Public Health Clinical Center Of Chengdu, ICU-2, Chengdu, China

**Keywords:** COVID-19, comorbidities, elderly, clinical characteristics, Sichuan Province

## Abstract

**Objectives:**

The co-occurrence of chronic diseases in the elderly is a common problem. However, the relationship between comorbidities and the prognosis of elderly patients with COVID-19 was not clear. This study was supposed to describe the clinical characteristics of elderly patients with COVID-19 infection from Sichuan province and the effects of comorbidity.

**Design:**

A retrospective study.

**Settings and participants:**

COVID-19 patients from Public Health Clinical Center of Chengdu between December 16, 2019 and February 26, 2020 were included in this study. Patients were divided into elderly group (≥60 years old) and non-elderly group (< 60 years old).

**Results:**

Elderly patients with COVID-19 indicated relatively higher proportion of comorbidities, and the most common were atherosclerotic cardiovascular disease (56.5%), hypertension (43.5%) and chronic pulmonary disease (21.7%). The proportion of severe cases was higher in elderly group than that in non-elderly group (73.9% and 42.2%, respectively, P=0.012). During hospitalization, elderly patients indicated relatively higher proportion of complications, such as shock (21.7%), respiratory failure (21.7%). The proportion of patients with a decreased number of CD8+ lymphocytes (82.6%) and B lymphocytes (77.8%) in elderly patients was significantly higher than that in non-elderly group (48.9% and 44.8%, respectively). All 3 deaths were elderly patients with comorbidities and the cell counts of T lymphocyte subsets, B and NK cells of them were significantly decreased at admission.

**Conclusions:**

Elderly patients with COVID-19 had a high proportion of severe cases and comorbidities, more likely to show low immune function, and indicate higher proportion of complications.

**Electronic Supplementary Material:**

Supplementary material is available for this article at 10.1007/s12603-020-1486-1 and is accessible for authorized users.

## Introduction

In December 2019, pneumonia of unknown cause broke out in Wuhan, China (1). The pathogen has been identified and was named as «COVID-19» in February 11, 2020 (2). Older populations are more susceptible to variety of diseases compared to younger, including COVID-19 infection (3, 4). Although the entire population is susceptible to the COVID-19, older patients had higher morbidity and case-fatality rate (5). The mortality rate (4.5%) of the people over 60 years old was much higher than that of the people under 60 years old (1.4%) (6). The co-occurrence of chronic diseases in the elderly is a common problem in the field of global public health (7). It was reported that more than half of the elderly in developed countries had more than three chronic diseases, meaning an individual suffers from two or more diseases with different pathology and no mutual dependence at the same time (8, 9). Studies in China such as Beijing and Shanghai suggest that more than 70 percent of elderly people in the community have two or more chronic diseases (10, 11). Compared with patients with a single disease, the hospitalization rate and fatality rate of patients with comorbidities are higher, and the clinical prognosis is significantly poor. However, the relationship of comorbidities and the prognosis of elderly patients with COVID-19 was not clear. Therefore, this study intended to describe the clinical characteristics of elderly patients with COVID-19 infection and the effects of comorbidity by comparing with non-elderly patients.

## Methods

### Participants

We retrospectively reviewed the medical records of patients COVID-19 who were hospitalized at Public Health Clinical Center of Chengdu, from December 16, 2019 to February 26, 2020. All confirmed cases were diagnosed as having 2019-nCoV pneumonia according to WHO interim guidance (12). Mild cases were defined as confirmed cases with fever, respiratory symptoms and radiographic evidence of pneumonia. Severe cases were defined as mild cases with one of the following conditions: (1) respiratory rate ≥ 30 times/min; (2) oxygen saturation ≤ 93% in resting state; (3) arterial oxygen partial pressure (PaO2)/oxygen concentration (FiO2) ≤300mmHg; (4) respiratory failure occurs and mechanical ventilation is required; (5) shock occurs; (6) other organ failure occurs and intensive care unit is required. Our study was approved by the ethics committee of West China hospital (No.2020/430) and the written informed consent was waived.

### Materials

The data on demographic, epidemiological, clinical, imaging and laboratory results, outcomes of patients included in this study were collected. All patients were categorized into two groups according to the age, the elderly group (≥60 years old) and the non-elderly group (< 60 years old). All collected data were compared between the two groups.

### Statistical analysis

All data were analyzed using the software SPSS version 20.0. Continuous measurements were represented as mean (^−^x ±s) and analyzed by t-test or median (P25 and P75) and analyzed by Mann-Whitney U test. χ^2^ test were used to analyze the classified variables. P value < 0.05 was considered to define statistical significance.

## Results

### The clinical and laboratory characteristics of elderly patients

68 patients who confirmed with COVID-19 infection were included in this study, aged from 19 to 88 years, and 35 (51.1%) male patients. Of the 68 patients, 45 (66.18%) and 23 (33.82%) were categorized into non-elderly group and elderly group, as shown in Table 1. In total, 62 (91.18%) patients had contact history. The most common symptoms in both groups were fever and cough.

**Table 1:** Baseline and clinical characteristics of COVID-19 patients

**Characteristics**	**Non-elderly patients**	**Elderly patients**	**P value**
Age, years	41 (31.5–49)	73 (64–81)	
*Sex*			*0.201*
Men	26 (57.8%)	9 (39.1%)	
Women	19 (42.2%)	14 (60.9%)	
History of contact	43 (95.6%)	19 (82.6%)	0.169
Comorbidities	23 (51.1%)	19 (82.6%)	0.017
Hypertension	4 (8.9%)	10 (43.5%)	0.003
Diabetes	5 (11.1%)	6 (26.1%)	0.464
Cardiovascular disease	9 (20.0%)	13 (56.5%)	0.005
Chronic pulmonary disease	0 (0.0%)	5 (21.7%)	0.003
Liver disease	6 (13.3%)	1 (4.3%)	0.409
Chronic kidney disease	1 (2.2%)	3 (13.0%)	0.109
*Signs and symptoms*			
Fever	38 (84.4%)	18 (78.3%)	0.522
Cough	34 (75.6%)	14 (60.9%)	0.364
Sputum production	8 (17.8%)	5 (21.7%)	0.750
Myalgia or fatigue	4 (8.9%)	1 (4.3%)	0.656
Dyspnoea	3 (6.7%)	1 (4.3%)	1.000
Chills	3 (6.7%)	1 (4.3%)	0.584
Temperature	37.12 (±0.62)	36.95 (±0.59)	0.281
Heart rate	89.44 (±13.67)	87.09 (±15.85)	0.526
Respiratory rate	20.93 (±2.88)	20.74 (±2.22)	0.778

The CT and laboratory tests of patients on admission were shown in Table 2. Most patients showed multiple lobe infection in both two groups, and no significant difference between this two groups (P=0.291). The red blood cell count (3.98x1012/L) and hemoglobin (122g/L) in the elderly group were lower than those in non-elderly group (4.64×1012/L and 138 g/L respectively). The C-reactive protein in the elderly group was higher than that in the young group (23.49 VS 9.93 mg/L, P = 0.047), but there was no significant difference in the procalcitonin between the two groups.

**Table 2:** CT and laboratory results of COVID-19 patients

**Characteristics**	**Non-elderly patients**	**Elderly patients**	**P value**
CT results			0.291
Multiple lobe lesion	35 (77.8%)	22 (95.7%)	
Right lobe lesion	7 (15.6%)	1 (4.3%)	
Left lobe lesion	2 (4.4%)	0 (0%)	
Normal	1 (2.2%)	0 (0%)	
*Laboratory indicators at admission*			
Leucocytes, × 10^9^ per L	5.37 (4.06–7.23)	5.86 (4.32–7.54)	0.841
Neutrophil count, × 10^9^ per L	3.26 (2.64–5.24)	4.19 (2.40–5.38)	0.795
Lymphocyte count, × 10^9^ per L	1.05 (0.65–1.62)	0.90 (0.51–1.46)	0.444
Neutrophils, %	69.50 (61.55–79.45)	74.10 (67.00–80.30)	0.437
Lymphocytes, %	22.40 (14.80–27.85)	17.00 (11.10–26.70)	0.396
Red blood cell count, × 10^12^ per L	4.64 (4.15–4.99)	3.98 (3.56–4.41)	0.001
Haemoglobin, g/L	138 (126–147)	122 (103–136)	0.002
Platelet count, × 10^9^ per L	175 (121–225)	154 (111–239)	0.382
Albumin, g/L	42.90 (37.75–46.10)	36 (32.95–40.93)	0.001
Alanine aminotransferase, U/L	27 (16–41)	19 (15–24)	0.082
Glutamyltransferase, U/L	27 (22–33.5)	30 (23–38)	0.479
Total bilirubin, umol/L	7.60 (5.25–14.80)	6.45 (3.65–8.13)	0.094
Lactate dehydrogenase, U/L	224 (200.5–274)	241 (205–296)	0.479
Creatine kinase isoenzymes, U/L	12 (9–17)	11 (9–17)	0.862
CD3 Count, cells/ul	666 (335.5–1022)	460 (318–721)	0.126
CD4 Count, cells/ul	396 (180–517)	313 (178–387)	0.270
CD8 Count, cells/ul	242 (137–334)	192 (111–219)	0.066
CD3 Percent	72 (63–78)	69 (66–75)	0.131
CD4 Percent	38 (31–46)	39 (34–45)	0.186
CD8 Percent	25 (22–32)	22 (17–30)	0.058
CD4/CD8 rate	1.48 (1.08–1.91)	1.79 (1.24–2.40)	0.126
B Count, cells/ul	93 (59–140)	68.50 (46.75–93.75)	0.073
NK Count, cells/ul	116 (59–159.5)	103 (53–166.5)	0.810
B Percent	11.37 (9.10–15.47)	9.80 (7.42–12.32)	0.118
NK Percent	13.76 (7.71–18.91)	15.57 (10.87–22.24)	0.143
Procalcitonin, ng/mL	0.023 (0.019–0.044)	0.028 (0.028–0.055)	0.664
C-reactive protein, mg/L	9.93 (4.22–22.95)	23.49 (6.95–57.94)	0.047
Plasma lactic acid, mmol/L	1.62 (1.30–2.13)	1.49 (1.20–1.81)	0.172

82.6% of the elderly patients showed a decrease in CD8+ lymphocyte count, which was much higher than that of in non-elderly group (48.9%), P = 0.009. 77.8% of patients showed the decrease of B lymphocyte in elderly group, which was also higher than that in the non-elderly group (44.8%) (P = 0.036). However, there was no significant difference in cell counts and rates of lymphocyte subsets and NK cells between the two groups, as shown in Table 3.

**Table 3:** Abnormal laboratory results in two groups of COVID-19 patients

**Characteristics**	**Non-elderly patients**	**Elderly patients**	**P value**
Leucocytes (× 10^9^ per L; normal range 3.5–9.5)			0.584
Increased	7 (15.6%)	3 (13.0%)	
Decreased	4 (8.9%)	4 (17.4%)	
Neutrophils (× 10^9^ per L; normal range 2–7)			0.961
Increased	7 (15.6%)	3 (13.0%)	
Lymphocytes (× 10^9^ per L; normal range 0.8–4)			0.422
Decreased	14 (31.1%)	10 (43.5%)	
CD3 Count (cells/ul; normal range 770–2041)			0.114
Decreased	26 (57.8%)	18 (78.3%)	
CD4 Count (cells/ul; normal range 414–1123)			0.065
Decreased	24 (53.3%)	18 (78.3%)	
CD8 Count (cells/ul; normal range 238–874)			0.009
Decreased	22 (48.9%)	19 (82.6%)	
B Count (cells/ul; normal range 90–560)			0.036
Decreased	13 (44.8%)	14 (77.8%)	
NK Count (cells/ul; normal range 150–1100)			1.000
Decreased	21 (72.4%)	13 (72.2%)	
C-reactive protein (mg/L; normal range 0–5)			0.389
Increased	32 (71.1%)	18 (81.8%)	
Alanine aminotransferase (U/L; normal range 0–37)			0.067
Increased	14 (31.1%)	2 (9.1%)	
Aspartate aminotransferase (U/L; normal range 0–37)			0.745
Increased	8 (17.8%)	5 (22.7%)	
Albumin (g/L; normal range 35–55)			0.052
Decreased	6 (13.3%)	8 (36.4%)	
Lactic dehydrogenase			0.594
Increased	16 (35.6%)	10 (45.5%)	
Hydroxybutyrate Dehydrogenase			0.193
Increased	18 (40.0%)	13 (59.1%)	
Creatine jubase			0.745
Increased	8 (17.8%)	5 (22.7%)	

### Comorbidities and the prognosis of elderly patients with COVID-19

The comorbidity of patients was shown in Table 1. Compared with non-elderly patients, old patients were more likely to combine with other basic diseases before infection of COVID-19 (P = 0.017). 13 (56.5%) elderly patients combined with atherosclerotic cardiovascular disease, and the proportion was higher than that in the non-elderly group (9 (20.0%), P = 0.005). The proportion of patients with hypertension was higher in elderly group than in non-elderly group (43.5% and 8.9%, respectively, P = 0.003). Five patients (21.7%) in the elderly group had chronic pulmonary disease, but no in the non-elderly group (P = 0.003).

The proportion of severe cases was higher in elderly group than that in non-elderly group (73.9% and 42.2%, respectively, P=0.012). During hospitalization, the proportion of elderly patients with respiratory failure was higher than that of in non-elderly patients (21.7% and 4.4%, respectively, P=0.039). Shock was also more common in older patients than non-elderly patients (21.7% vs 2.2%, P=0.015). And of the 6 patients with shock, 5 had a history of atherosclerotic cardiovascular disease. The proportion of elderly patients requiring mechanical ventilation during treatment was higher than that of younger patients (30.4%, 8.9%, respectively, P=0.020). As shown in table 4, the prognosis of the elderly patients was worse than that of the non-elderly group (P=0.006). All 3 deaths were elderly patients with underlying diseases before admission. The level of C-reactive protein of these dead patients was significantly increased and the cell counts of CD3+, CD4+, and CD8+ T lymphocytes, B lymphocytes and NK cells were significantly decreased at admission, as shown in supplementary table 1.

**Table 4:** Prognosis of COVID-19 patients

**Characteristics**	**Non-elderly patients**	**Elderly patients**	**P value**
Severe cases	19 (42.2%)	17 (73.9%)	0.012
Complications			
Acute liver and kidney injury	17 (37.8%)	5 (21.7%)	0.274
Coagulation function abnormal	0 (0%)	3 (13.0%)	0.035
Respiratory failure	2 (4.4%)	5 (21.7%)	0.039
Shock	1 (2.2%)	5 (21.7%)	0.015
Oxygen support			0.020
Low-flow nasal cannula	36 (80.0%)	11 (47.8%)	
High-flow nasal cannula	5 (11.1%)	5 (21.7%)	
Mechanical	4 (8.9%)	7 (30.4%)	
Prognosis			0.006
Discharge	36 (80.0%)	13 (56.5%)	
Hospital readmission	4 (8.9%)	0 (0.0%)	
Hospitalisation	5 (11.1%)	7 (30.4%)	
Death	0 (0.0%)	3 (13.0%)	
Days from illness onset to hospital admission	7 (4–11.5)	7 (2–8)	0.711
Hospitalization days	9 (7–12)	10 (8–14)	0.915

## Discussion

This study collected the clinical information of 68 patients with COVID-19 infection who hospitalized in Public Health Clinical Center of Chengdu, Sichuan. The clinical characteristics of elderly patients with COVID-19 were descripted by compared with non-elderly patients.

We observed more patients with COVID-19 were men which was same as the results of previous study (13–15). The proportion of elderly patients was 33.82%, which was similar to other studies (16, 15). In our study, the most common symptom were fever (78.3%) and cough (60.9%) in elderly patients, which was similar to other researches (17–19). A previous study found that the incidence of multilobe lesions in elderly patients was significantly higher than in non-elderly patients (16). We also observed the higher incidence of multilobe lesions in elderly patients, although no statistically difference.

In this study, the proportion of patients with decreased leucocytes count was 11.8%, lymphocytes 35.3%, lower than that of in a previous study (33.7% and 83.2%, respectively) (20). The proportion of patients with a decreased number of CD8+ T lymphocytes and B lymphocytes in elderly patients was significantly higher than that in non-elderly group, suggesting that elderly patients were more likely to indicate low immune function. The previous study observed that the acute phase of SARS in humans was associated with a severe reduction in the number of T cells in the blood (21). The level of C-reactive protein in elderly patients was significantly higher than that in the non-elderly group, which was consistent with previous study and similar to MERS-CoV infection (22, 16).

We found that the prognosis of patients with COVID-19 in elderly patients was worse, which was in accordance with the results of other studies (23, 24, 16). All 3 dead cases were elderly patients, and had multi-system disease before infected by COVID-19, with decreased umber of T lymphocyte subsets, B lymphocytes and NK cells. An investigation of 463 patients with COVID-19 disease revealed the decreased amount of total lymphocytes, T lymphocyte subsets in the severe type patients (25). Therefore, patients with COVID-19 should actively deal with their comorbidities, prevent bacterial infection and strengthen immune support treatment.

This study found that older patients with COVID-19 indicated relatively higher proportion of comorbidities than non-elderly patients, and the most common comorbidities were atherosclerotic cardiovascular disease (56.5%), hypertension (43.5%) and chronic pulmonary disease (21.7%), which was consistent with other studies (26, 15). These multiple disease coexisting in elderly patients affected each other and leaded to complicated and complex diseases. Severe patients were significantly more in elderly patients than non-elderly patients, which was in accordance with previous researches (27, 19). Comorbidities was a risk factor for severe cases (OR=2.95, P=0.035). We also observed that elderly patients indicated relatively higher proportion of complications, such as shock (21.7%). And of the 6 patients with shock, 5 had a history of atherosclerotic cardiovascular disease. Therefore, actively dealing with the complications may improve the prognosis of patients.

The co-occurrence of chronic diseases in the elderly is a common problem in the field of global public health (7). It was reported that more than half of the elderly in developed countries had more than three chronic diseases, meaning an individual suffers from two or more diseases with different pathology and no mutual dependence at the same time (8, 9). Studies in China such as Beijing and Shanghai suggest that more than 70 percent of elderly people in the community have two or more chronic diseases (10, 11). Compared with patients with a single disease, the hospitalization rate and fatality rate of patients with comorbidities are higher, and the clinical prognosis is significantly lower. A cohort study showed that comorbidities were independent predictors of clinical prognosis in patients with cardiovascular disease (28). Comorbidities make medical decisions more complex and difficult. And, comorbidities often involve multiple medications, and the interactions between drugs and diseases often lead to worse final efficacy, worse prognosis, more adverse reactions and more medical costs (28).

At present, there are no guidelines for comorbidity management. In 2012, the American geriatrics society organized an expert group to formulate the guiding principles for clinical management of comorbidity in the elderly, such as considering the complexity and feasibility of the treatment plan; optimize the treatment plan to choose the one that benefits the most, does the least damage and can improve the quality of life, and carry on the elaboration explanation one by one (9). Doctors should be reminded that the treatment of comorbidities should emphasize patient-centered treatment, considering the whole patient and giving the most appropriate individual treatment.

### Limitations

There are some limitations in our study. First, not all COVID-19 cases in Sichuan were enrolled in this study, but only patients admitted to Public Health Clinical Center of Chengdu. And the sample size of our study is relatively small. A study which cover wide population is needed to get more accurate results. Secondly, more detailed patient information was not analyzed, especially different treatment methods and their outcomes.

## Conclusions

In this study we observed elderly patients infected with COVID-19 had a high proportion of severe cases and comorbidities, more likely to show low immune function and indicated higher proportion of complications during the course of COVID-19 infection. All dead cases were elderly patients and with low immunity and comorbidities. Therefore, we should pay more attention to elderly patients, especially their comorbidities, and try to give the most appropriate individual treatment for older patients with COVID-19 infection.

## Electronic Supplementary Material

Supplementary Table 1 Characteristics of dead patients with COVID-19

## Data Availability

*Availability of data and material:* The datasets used and/or analyzed during the current study are available from the corresponding author on reasonable request.

## Notes

*Ethics approval/Informed consent:* Our study was approved by the ethics committee of West China hospital (No.2020/430) and the written informed consent was waived.
