# Supplementary material for: Effects of Comorbidities on the Elderly Patients with COVID-19: Clinical Characteristics of Elderly Patients Infected with COVID-19 from Sichuan, China
Source: J Nutr Health Aging. 2020 Oct 12;25(1):18–24. doi: 10.1007/s12603-020-1486-1 (PMC7548532; doi:10.1007/s12603-020-1486-1)
Supplement: Supplementary file 1 — Supplementary Table 1 Characteristics of dead patients with COVID-19 [file 12603_2020_1486_MOESM1_ESM.doc]

Supplementary Table 1 Characteristics of dead patients with COVID-19

| Patient number | 1 | 2 | 3 |
| --- | --- | --- | --- |
| Age, years | 64 | 73 | 80 |
| Sex | female | male | female |
| Medical history | pulmonary fibrosis, diabetes, diabetic nephroopathy | hypertention, coronary disease, chronic kidney disease | hypertention, coronary disease |
| C-reactive protein (mg/L) | 174.17 | 23.49 | 60.3 |
| CD3 Count (cells/ul) | 209 | 168 | 229 |
| CD4 Count (cells/ul) | 80 | 105 | 145 |
| CD8 Count (cells/ul) | 122 | 44 | 74 |
| B Count (cells/ul) | 2 | 23 | 36 |
| NK Count (cells/ul) | 23 | 60 | 50 |
